# Multimodal characterization of cortical hyperexcitability as a driver of cognitive decline in neurocognitive disorders: study protocol for the LENDÜLET Neurocognitive Research Project

**DOI:** 10.3389/fnagi.2026.1809381

**Published:** 2026-08-14

**Authors:** András Attila Horváth

**Affiliations:** 1 Neurocognitive Research Centre, Nyírõ Gyula National Institute of Psychiatry and Addictology, Budapest, Hungary; 2 Department of Anatomy, Histology and Embryology, Semmelweis University, Budapest, Hungary; 3 Institute of Cognitive Neuroscience and Psychology, HUN-REN Research Centre for Natural Sciences, Budapest, Hungary; 4 Faculty of Medicine, University of Lisbon, Lisbon, Portugal

**Keywords:** attention deficit hyperactivity disorder, autism spectrum disorder, electroencephalography, functional MRI, hyperexcitability, mild cognitive impairment, subclinical epileptiform activity

## Abstract

**Introduction:**

Cognitive impairment is a major source of disability in neurodegenerative conditions, and it is also highly prevalent in autism spectrum disorder and attention-deficit/hyperactivity disorder. Across these conditions, subclinical epileptiform activity and resting-state functional hyperactivity have been repeatedly described, suggesting a shared state of cortical hyperexcitability. The LENDÜLET Neurocognitive Research Project aims to determine the incidence and multimodal characteristics of cortical hyperexcitability in multiple patient populations and to elucidate how hyperexcitability relates to cognitive performance, large-scale network connectivity, sleep-dependent memory consolidation, and tau/amyloid burden.

**Methods:**

In a prospective, multimodal observational study, we are recruiting 75 participants per group (patients with mild cognitive impairment, patients with autism spectrum disorder, patients with attention deficit/hyperactivity disorder, and healthy controls; total *N* = 300) at the National Institute of Psychiatry and Addictology (Budapest, Hungary). All participants are undergoing a harmonized diagnostic protocol including detailed neuropsychological assessment, structural MRI, resting-state functional MRI, and 24-h ambulatory electroencephalography. Patients with neurodegenerative conditions will additionally undergo CSF sampling. Individuals who exhibit markers of cortical hyperexcitability will be invited to an in-ward mechanistic substudy (3-day video-electroencephalography monitoring with repeated neuropsychological paradigms, overnight sleep recordings, and spindle analysis, and serial blood sampling to characterize circadian dynamics of tau and amyloid.

**Anticipated results:**

Primary outcome is the incidence and distribution of cortical hyperexcitability in each diagnostic group relative to controls. Secondary outcomes include relationships between hyperexcitability and (a) structural atrophy and white matter integrity; (b) functional connectivity within and between default mode, salience, and attention networks; (c) cerebrospinal and plasma tau/amyloid levels; and (e) domain-specific cognitive performance. The current paper describes the study design, while the results are not reported.

**Discussion:**

This study will provide the first systematic, multimodal assessment of cortical hyperexcitability across multiple neurocognitive disorders with shared vulnerability to epilepsy and cognitive decline. The resulting biomarker panel may support risk stratification, inform clinical trial design for anti-hyperexcitability interventions, and ultimately enable individualized prevention strategies for cognitive decline.

## 1 Introduction

### 1.1 Rationale

Cognitive deterioration is a core feature of major neurocognitive disorders (NCD), particularly mild cognitive impairment (MCI) and Alzheimer’s disease (AD), but is also highly prevalent in autism spectrum disorder (ASD) and attention-deficit hyperactivity disorder (ADHD) (Leffa et al., 2023; Torenvliet et al., 2023). The cumulative prevalence of these conditions approaches 20% in developed societies, imposing a substantial medical, social, and economic burden. While these disorders differ in clinical presentation and classical neuropathology, converging evidence suggests that aberrant neuronal excitability and network dysfunction may represent a shared pathogenic pathway (Horvath et al., 2020).

In ASD, cortical hyperexcitability results from an imbalance between excitatory glutamatergic and inhibitory GABAergic signaling, leading to sensory hypersensitivity and seizure susceptibility (Bozzi et al., 2018; Takarae and Sweeney, 2017). Similarly, ADHD studies demonstrate altered excitatory-inhibitory control, often associated with hyperdopaminergic and glutamatergic network activity that disrupts attentional regulation and impulse control (Bauer et al., 2018). Over time, persistent neuronal hyperactivity may drive metabolic stress, calcium dysregulation, and oxidative damage, processes also observed early in neurodegenerative diseases such as Alzheimer’s (Muddapu et al., 2020). In AD, cortical and hippocampal hyperactivity are early features that progress to hypoactivity and neuronal loss as degeneration advances (Busche and Konnerth, 2015). Shared molecular pathways, including dysregulated Wnt/mTOR signaling and aberrant synaptic plasticity, further connect these conditions, suggesting a continuum from neurodevelopmental to neurodegenerative pathology (Grünblatt et al., 2023). Impaired glymphatic clearance and cerebrospinal fluid flow, as proposed in ASD and AD, may exacerbate excitability by allowing toxic protein accumulation (Phillips et al., 2025). In addition to mechanistic observations, the chronic excitatory state was also confirmed by clinical biomarkers in these disorders.

Patients with MCI, ASD, and ADHD frequently exhibit epileptiform discharges on electroencephalography (EEG) recordings, resting-state hyperactivity on functional magnetic resonance imaging (fMRI), and lower cortical excitability thresholds measured with transcranial magnetic stimulation (Takarae and Sweeney, 2017; Targa Dias Anastacio et al., 2022; Ugarte et al., 2023). In AD, subclinical epileptiform activity (SEA)—epileptiform discharges without overt clinical seizures—is increasingly recognized as a major contributor to accelerated cognitive decline (Kamondi et al., 2024; Vossel et al., 2026). Long-term EEG studies have demonstrated that SEA in AD is associated with faster cognitive deterioration and specific neuroanatomical substrates, including early precuneus thinning and larger general cortical volumes (Horvath et al., 2019; Nous et al., 2024). Previous observations have shown that sleep EEG is particularly sensitive to detect epileptiform activity in dementia (Horváth et al., 2017; Nous et al., 2024; Vossel et al., 2016). Some studies also suggest that SEA in dementia is associated with a special, probably more aggressive neuroimaging phenotype of AD (Cretin et al., 2016). Earlier reports have also demonstrated that SEA is linked to more rapid progression of cognitive decline in AD (Horvath et al., 2021; Vossel et al., 2013, 2016).

Beyond AD, SEA and other markers of cortical hyperexcitability are reported in ASD and ADHD, where they may relate to attentional deficits, learning problems, and behavioral disturbances (Lee et al., 2016; Spence and Schneider, 2009). Functional MRI studies in these populations describe hyperactivity and altered connectivity within key large-scale networks, including the default mode network (DMN), salience network (SN), and attention networks (AN), particularly early in the disease course (Hull et al., 2017; Konrad and Eickhoff, 2010). Some recent observations also suggest that the observed hyperactivity and connectivity changes may reflect an early excitatory/inhibitory (E/I) imbalance in preclinical AD (Fortel et al., 2023; Li et al., 2025; Ranasinghe et al., 2025). Based on these reports, some authors propose that multilevel changes in neural networks may be associated with a very early shift in E/I balance, a feature of AD pathology (Stam et al., 2023). A similar link between hyperactivity and hyperexcitability has been proposed in ASD (Port et al., 2019) and ADHD (Mamiya et al., 2021). However, disease-specific spatial differences in E/I imbalance have also been proposed as the primary involvement of the prefrontal cortex in ASD and ADHD (Ajram et al., 2017), and the DMN-specific imbalance in preclinical and prodromal AD (Giorgio et al., 2024). These commonalities suggest that ASD and ADHD may represent early-life manifestations of circuit-level dysregulation, while Alzheimer’s reflects its late-life degeneration (Pievani et al., 2011).

Despite this emerging evidence, several major knowledge gaps remain (Kamondi et al., 2024; Vossel et al., 2026): 1) There is no systematic, multimodal characterization of SEA and fMRI hyperactivity across MCI, ASD, ADHD and healthy aging within a unified protocol; 2) It is unclear to what extent hyperexcitability markers predict domain-specific cognitive impairment and neurodegeneration (atrophy, white matter damage); 3) The relationship between hyperexcitability, large-scale functional connectivity, sleep structure, sleep spindles, and memory consolidation is poorly understood; 4) Whether hyperexcitability modulates the burden or circadian dynamics of tau and amyloid-β pathology, particularly in prodromal AD, is unknown.

Addressing these gaps is essential for refining risk-stratification strategies for individuals at risk of cognitive decline and for designing targeted therapeutic approaches to modulate cortical excitability (Kamondi et al., 2024). In this paper, we highlight a study design to address these gaps, while the results of the ongoing study are not reported.

### 1.2 Objectives and hypotheses

The overarching objective of the LENDÜLET Neurocognitive Research Project is to determine whether cortical hyperexcitability represents a common, mechanistically relevant substrate linking epileptiform activity to cognitive decline in aging and neurodevelopmental disorders. The LENDÜLET Research Grant is the most prestigious scientific grant of the Hungarian Academy of Sciences, awarded to establish individual working groups dedicated to research topics of extraordinary importance to public health and society’s strengths. We structure the project into two main work packages (outward clinical multimodal study, and in-ward mechanistic substudy), with the following specific aims and hypotheses.

Our working hypotheses are the following:

1. **Incidence of cortical hyperexcitability.**
2. *Hypothesis 1:* The incidence of cortical hyperexcitability (SEA on EEG, network-specific hyperactivity on resting-state fMRI) is significantly higher in MCI, ASD, and ADHD than in cognitively healthy controls.
3. **Association with cognitive performance.**
4. *Hypothesis 2:* Higher levels of cortical hyperexcitability are associated with poorer performance across standard neuropsychological measures of memory, attention, executive function, and visuospatial skills.
5. **Association with structural brain changes.**
6. *Hypothesis 3:* Hyperexcitability is associated with a distinct pattern of cortical volumes and white matter alterations on structural MRI, particularly in hippocampal, medial temporal, and parietal regions.
7. **Association with functional connectivity.**
  *Hypothesis 4:* Hyperexcitability is associated with altered resting-state functional connectivity within and between DMN, SN, and AN, indicative of maladaptive network reorganization.
8. **Concordance across modalities.**
9. *Hypothesis 5:* There is significant cross-correlation between EEG-detected SEA and fMRI hyperactivity within the same individual.
10. **Interaction with tau and amyloid pathology (MCI/AD continuum).**
11. *Hypothesis 6:* In MCI, higher cortical hyperexcitability is associated with distinct CSF and plasma levels of tau and amyloid-β (Aβ40, Aβ42, t-tau, p-tau181) and with an unfavorable Aβ42/Aβ40 ratio.
12. **Impact on memory maintenance and consolidation.**
  *Hypothesis 7:* Hyperexcitability is associated with impaired short-term retention and overnight consolidation of verbal memory.
13. **Impact on sleep architecture and spindle dynamics.**
14. *Hypothesis 8:* Hyperexcitability is associated with disrupted sleep architecture, altered N2/N3 power spectra, and reduced or disorganized slow and fast sleep spindles, which in turn mediate effects on memory consolidation.

## 2 Methods

### 2.1 Study design

**FIGURE 1:**
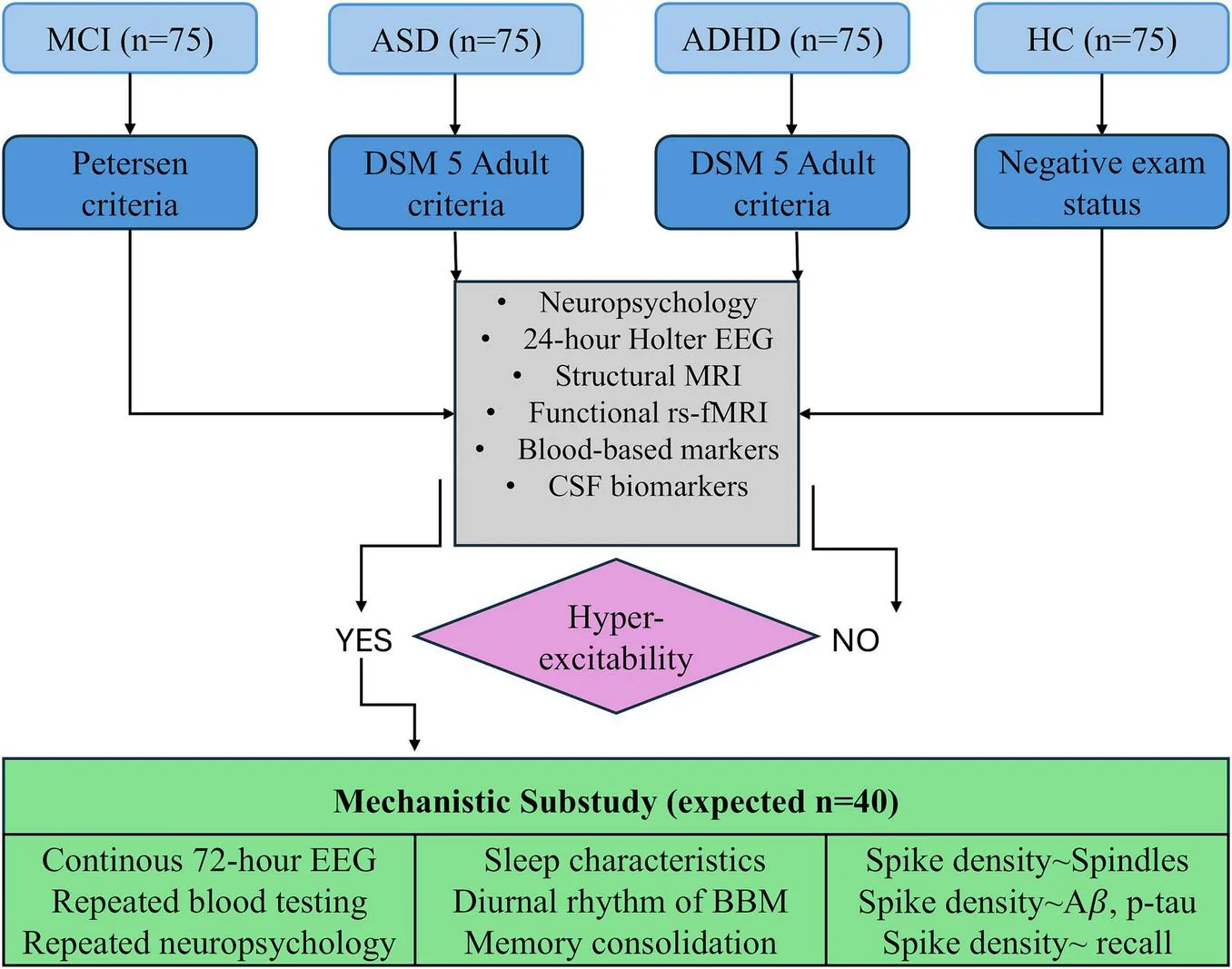
Overview of the LENDÜLET Neurocognitive Research Project design and workflow. MCI, mild cognitive impairment; ADHD, attention deficit/ hyperactivity disorder; ASD, autism spectrum disorder; HC, healthy control; DSM, Diagnostic and Statistical Manual of Mental Disorders, 5th Edition; EEG, electroencephalography; MRI, magnetic resonance imaging; rs, resting-state; fMRI, functional magnetic resonance imaging; CSF, cerebrospinal fluid; BBM, blood-based biomarkers; Aβ, amyloid-beta.

This is a prospective, single-center, multimodal observational study being conducted at the Nyírö Gyula National Institute of Psychiatry and Addictology, led by the Neurocognitive Research Centre (NRC) in Budapest, Hungary. The study began in 2023 and is expected to terminate in 2028. The project comprises a cross-sectional, multimodal phenotyping of four groups (MCI, ASD, ADHD, and healthy controls; *N* = 75 per group) with standardized neuropsychological testing, structural MRI, resting-state fMRI, 24-h ambulatory EEG, and blood/CSF biomarkers. We are also performing a nested mechanistic substudy in a subset of participants across all groups who exhibit markers of cortical hyperexcitability. These participants are undergoing a 3-day in-ward protocol with continuous video-EEG, repeated neuropsychological testing, sleep staging and spindle analysis, and serial blood sampling. The study duration is 5 years, with the first 3 years focused on recruitment and baseline assessments, and the subsequent years emphasizing mechanistic analyses and dissemination. The protocol is summarized in Figure 1.

The proposed design is a pooled cross-diagnostic study protocol involving three patient populations and integrating data across these groups to identify shared and distinct neurobiological mechanisms. This study design has drawn increasing attention in the literature on neuropsychiatric disorders (Kan et al., 2023; Wigman et al., 2015). We are recruiting participants based on established diagnostic criteria, alongside age- and sex-matched healthy controls. Clinical, cognitive, and neuroimaging data will be harmonized across cohorts, ensuring comparable measures of executive function, memory, and social cognition. Structural and functional MRI, as well as EEG, are assessing neural network connectivity, focusing on regions implicated in excitation/inhibition (E/I) balance, such as the prefrontal cortex, hippocampus, and sensory cortices. We are using additional assays, including plasma and cerebrospinal fluid biomarkers, capturing molecular signatures of synaptic regulation and inhibitory neurotransmission. This approach enables identification of transdiagnostic neural signatures that transcend categorical diagnoses, offering a framework for understanding common circuit dysfunctions underlying both neurodevelopmental and neurodegenerative conditions.

### 2.2 Participants

#### 2.2.1 Eligibility criteria

Four groups are in recruitment:

- MCI (older adults at risk for AD):
  Age: ≥ 55 years
  Meeting Petersen’s criteria for MCI: subjective cognitive complaint corroborated by an informant, objective impairment in at least one cognitive domain, preserved basic activities of daily living, absence of dementia (Petersen, 2016).
  Supporting evidence from structural MRI and neuropsychology.

- ASD:
  Adult participants fulfilling DSM-5 criteria for ASD, based on standardized clinical assessment and validated instruments (Murphy et al., 2016).

- ADHD:
  Adult participants fulfilling DSM-5 criteria for ADHD, based on structured clinical assessment and established rating scales (Young and Goodman, 2016).

- Healthy controls (HC):
  No subjective cognitive complaints.
  Normal neurological examination and neuropsychological performance.
  Normal structural MRI and routine laboratory tests.
  No history of major psychiatric or neurological disease.

#### 2.2.2 General exclusion criteria

Applied across all groups to reduce confounders:

- History of central nervous system infection, significant brain lesions (stroke, extensive small-vessel disease, white matter infarcts), demyelinating disease, hydrocephalus, or major head trauma with loss of consciousness.
- Untreated vitamin B12 deficiency, hypothyroidism, syphilis, or HIV infection.
- Major depressive episode, schizophrenia, intellectual disability, or current substance dependence.
- Renal or hepatic failure, or other serious systemic illness likely to affect cognition.
- Current use of psychoactive medications significantly affects EEG/fMRI/neuropsychology (e.g., high-dose benzodiazepines, barbiturates), unless the dose is stable and deemed acceptable by investigators.
- Established epilepsy or history of unprovoked seizures
- Standard contraindications to MRI (e.g., ferromagnetic implants, pacemaker, unstable cardiac disease).

#### 2.2.3 Recruitment and consent

We are recruiting from:

- Outpatient clinics of the National Hospital for dementia/MCI, ASD, and ADHD.
- In-house patient registries, including the AlzEpi Cohort Observational Library (ACOL).
- Collaborating with outpatient neurology and psychiatry services.
- Patient organizations and community outreach.

Potentially eligible individuals are receiving written and verbal information about the study. Written informed consent will be obtained from all participants (or legally authorized representatives if required) prior to any study-specific procedures. The entire study, including recruitment, data acquisition, data analysis, and reporting, will be carried out by the same team (neurologist, neuropsychologist, neurophysiologist, MRI assistant, radiologists, and clinical researchers) throughout.

#### 2.2.4 Sample size justification

Power calculations for the primary cross-sectional study were informed by previous investigations of cortical hyperexcitability and subclinical epileptiform activity (SEA) in neurocognitive disorders [summarized by Horvath et al. (2020)]. Assuming a medium effect size (Cohen’s *d* = 0.5), a two-sided significance level of α = 0.05, and four diagnostic groups, a sample size of 75 participants per group (total *N* = 300) provides more than 90% power to detect clinically meaningful between-group differences in the primary outcomes, including the incidence of SEA and resting-state fMRI hyperactivity.

The mechanistic in-ward substudy is enrichment-based and will include participants demonstrating evidence of cortical hyperexcitability. Assuming a conservative prevalence of approximately 15% across the clinical groups, we anticipate recruiting approximately 45 eligible participants, with an expected final sample of approximately 40 after accounting for an estimated 20% attrition rate.

The mechanistic substudy is designed primarily to characterize within-subject physiological relationships among cortical hyperexcitability, sleep architecture, memory consolidation, and circulating biomarkers using repeated-measures analyses. Because repeated observations substantially increase statistical efficiency for within-subject comparisons, this sample size is considered appropriate for estimating effect sizes, assessing feasibility, and identifying candidate mechanistic pathways. In contrast, more analytically demanding models, including formal mediation analyses and subgroup comparisons across diagnostic categories, are expected to have limited statistical power with the anticipated sample size. Accordingly, these analyses will be considered exploratory and hypothesis-generating, with emphasis placed on effect size estimation, confidence intervals, and model uncertainty rather than statistical significance alone. Findings from these exploratory analyses will inform sample size calculations and analytic strategies for future confirmatory multicenter studies.

### 2.3 Study procedures for prospective cross-sectional analysis

All participants are underogoing the following within 3 months:

1. Clinical neurological and psychiatric examination.
2. Comprehensive neuropsychological assessment.
3. Structural and resting-state fMRI.
4. 24-h ambulatory (Holter) EEG.
5. Blood sampling (all groups) and CSF sampling (MCI subset, where clinically indicated).

Data are stored in an anonymized research database (ACOL).

#### 2.3.1 Neuropsychological assessment

Neuropsychological testing (Hungarian language versions) will be performed by trained neurologists or neuropsychologists using a unified UDS3-based battery (Boccardi et al., 2022). Instruments include:

- Global cognition: Addenbrooke Cognitive Examination–Revised (ACE-R), Mini-Mental State Examination (MMSE).
- Memory and learning: Rey Auditory Verbal Learning Test (RAVLT: Sum of first five trials [Sum5], delayed recall after 30 min [Trial 7]).
- Attention and executive functions: Trail Making Test A (TMT-A: processing speed) and B (TMT-B: set-shifting).
- Visuospatial and constructional skills: Benson Figure Copy (immediate copy and delayed recall).
- Working memory: Number Span Backward (NSB).
- Language: Category (semantic) and phonemic verbal fluency.
- Mood and anxiety: Beck Depression Inventory-II (BDI-II), Spielberger State and Trait Anxiety Inventory (STAI-S, STAI-T).

Cut-offs for normal performance (e.g., MMSE > 25, age- and education-adjusted norms) will be applied for diagnostic purposes and covariate control. This battery has been validated and widely used by our group in previous cohorts (Berente et al., 2022; Csukly et al., 2016; Csukly et al., 2024; Unoka et al., 2026).

#### 2.3.2 Structural MRI

All MRI examinations will be conducted on a 3T Siemens Magnetom Verio scanner using a 12-channel head coil. The protocol includes:

- T1-weighted 3D MPRAGE (TR 2,300 ms; TE 3.4 ms; voxel 1 × 1 × 1 mm).
- Additional high-resolution T1-weighted 3D spoiled gradient echo (T1W 3D Turbo Field Echo; TR 9.7 ms; TE 4.6 ms; voxel 1 × 1 × 1 mm).
- T2-weighted, FLAIR, and 64-direction diffusion tensor imaging (DTI) sequences to evaluate lesions and white matter integrity.

Cortical reconstruction and volumetric segmentation will be performed using FreeSurfer 6.0 (“recon-all” pipeline with default parameters). Quality-checked outputs will provide cortical thickness and volume estimates in standard parcellations. DTI-based structural connectivity indices will be derived using established pipelines. The exact pipeline is described in previous studies published by our group (Bolla et al., 2025; Csukly et al., 2016).

#### 2.3.3 Resting-state fMRI

Resting-state fMRI will be acquired using T2*-weighted echo-planar imaging (EPI) sequences (TR 2,000 ms; TE 30 ms; flip angle 70–79°; voxel 3 × 3 × 3–4 mm; ∼10-min duration). Participants will be instructed to remain still, keep their eyes closed (or fixate on a cross, as per the scanner-specific protocol), and avoid falling asleep.

Preprocessing and functional connectivity analysis will be performed with the CONN toolbox (MATLAB) using a standard pipeline:

- Realignment and unwarping; slice-timing correction.
- Outlier detection (ART-based scrubbing).
- Direct segmentation and normalization (GM/WM/CSF, MNI space).
- Spatial smoothing.
- Band-pass filtering (0.008–0.09 Hz).
- Regression of white matter, CSF, and motion parameters.

Regions of interest (ROIs) will be defined *a priori* within the DMN, SN, and AN based on the literature and group consensus. Seed-based connectivity (SBC) analyses will compute Fisher-z-transformed correlations between ROI time series and voxel-wise signals. Age and sex will be included as covariates. Group differences (e.g., HC > MCI, HC > ASD, HC > ADHD) will be evaluated using voxel-wise ANCOVA with cluster-level FDR correction (cluster pFDR < 0.05; voxel *p* < 0.001 uncorrected). Hyperactivity will be defined as significantly elevated connectivity or activity within predefined network ROIs of DMN, SN, and AN relative to HC normative values obtained from our reference database (ACOL). For further details, see section “2.4.1 Participant selection and extraction of normative values.” Participants will be categorized as fMRI-positive or fMRI-negative for cortical hyperexcitability. The exact fMRI pipeline is characterized in previous studies published by our group (Bolla et al., 2023, 2025).

#### 2.3.4 Ambulatory EEG (Holter-EEG)

Participants are undergoing a 24-h, 19-channel ambulatory EEG (Micromed Morpheus, 10–20 system). Key parameters:

- Bipolar longitudinal montage; sensitivity 10 μV/mm; speed 30 mm/s.
- Filters: 0.5–70 Hz band-pass, 50 Hz notch.
- Additional electrooculogram leads for sleep staging.

The conventional 19-channel scalp EEG (international 10–20 system) was selected because it represents the current clinical standard for prolonged ambulatory EEG in patients with cognitive disorders and provides an optimal balance between spatial coverage, patient comfort, recording stability, and feasibility during 24-h outpatient monitoring. While high-density EEG offers improved spatial localization of cortical sources, its practical advantages for prolonged ambulatory recordings remain limited because of increased preparation time, electrode instability, patient burden, and susceptibility to movement artifacts. As the primary objective of the EEG component is the detection and quantification of subclinical epileptiform activity rather than source localization, the standard 19-channel montage is considered appropriate and consistent with previous studies from our group and the broader dementia literature (Horváth et al., 2017, 2018b; Horvath et al., 2021).

The recording bandpass of 0.5–70 Hz was selected to preserve conventional clinical EEG frequencies while minimizing slow baseline drift and high-frequency muscle artifacts. The lower cutoff (0.5 Hz) retains physiologically relevant slow-wave activity required for sleep staging, whereas the upper cutoff (70 Hz) preserves the frequency content of epileptiform spikes and sharp waves while reducing contamination from electromyographic activity. Epileptiform discharges will be defined according to the International Federation of Clinical Neurophysiology recommendations (Kane et al., 2019) as transient spikes or sharp waves with durations of 20–200 ms, followed by an associated slow wave, when present. This definition will facilitate comparison with previous clinical neurophysiology studies and established diagnostic practice.

All recordings will be independently reviewed by two experienced clinical neurophysiologists who are blinded to diagnosis and other clinical information. Epileptiform discharges will be accepted only when both reviewers reach consensus. Prior to consensus adjudication, inter-rater agreement will be quantified using Cohen’s κ coefficient for the presence or absence of subclinical epileptiform activity, with κ values and 95% confidence intervals reported in the final study results. Discordant cases will be reviewed jointly to establish the final classification. Automated spike detection (Persyst) will serve as a supporting tool for quantifying spike density, amplitude, and duration, while all final classifications remain based on expert visual assessment. The average spike density (spikes/hour) will be computed, and scalp distribution will be categorized (frontal, temporal, parietal, occipital, etc.) using Micromed SystemPLUS 98. Participants will be labeled as EEG-positive or negative for SEA relative to HC normative values obtained from our reference database (ACOL). The SEA detection pipeline is reported in our previous publications (Horváth et al., 2018b; Horvath et al., 2021).

#### 2.3.5 CSF and blood biomarkers

All participants will provide blood samples for plasma biomarkers. MCI participants undergoing clinically indicated lumbar puncture will provide CSF samples. Samples will be stored at -80°C and analyzed using validated ELISA assays for:

- Aβ40, Aβ42.
- Total tau (t-tau).
- Phospho-tau181 (p-tau181).

CSF biomarkers will be interpreted in the context of AD-related pathology; blood biomarkers will support mechanistic analyses and may inform future non-invasive stratification (Bouwman et al., 2022).

### 2.4 Study protocol for mechanistic in-ward testing

#### 2.4.1 Participant selection and extraction of normative values

Across all groups (HC, MCI, ASD, ADHD), participants with clear evidence of cortical hyperexcitability—defined by at least one of: SEA on EEG above a predefined density threshold; or fMRI-defined hyperactivity in DMN/SN/AN—are invited to participate. We will initially prioritize HC and MCI subjects during the pilot phase to refine the in-ward protocol, then extend to ASD/ADHD.

Normative values for the fMRI and EEG metrics will be calculated using the ACOL dataset. The ACOL dataset is our clinical registry, founded in 2014, and contains longitudinal data from 500 individuals at various stages of the cognitive continuum. The dataset includes 280 healthy individuals (mean age = 55) with records on clinical anamnesis, demographics, blood-based biomarkers, neurological examination, medications, neuropsychology, 24-h Holter-EEG, 10-min resting EEG, sMRI, 10-min resting fMRI, pupillometric data, fine-movement analysis, and autonomic functions. All participants have been scanned with the same MRI scanner (Siemens Magnetom 3T) and examined with the same EEG recording system (Micromed Morpheus Holter-EEG). Further details on the dataset can be found at the EuroFingers Consortium website^1^.

To generate normative fMRI values, we perform rigorous preprocessing of all scans—motion correction, spatial normalization, smoothing, and signal denoising—to ensure comparability across individuals. Next, functional metrics, including functional connectivity strength, regional homogeneity (ReHo), amplitude of low-frequency fluctuations (ALFF), Hurst-exponent, and network efficiency, are extracted for each subject. Within the healthy control group, these measures are then aggregated and standardized by computing the mean and standard deviation of each metric across all participants to generate z-score distributions that define the “normal” range. Statistical modeling (Gaussian mixture) is used to identify normative intervals and detect outliers. To account for biological variability, the dataset is stratified by relevant covariates such as age, sex, and head motion. The resulting normative models can then serve as a statistical reference framework, allowing patient metrics to be expressed as deviation scores (z-scores) that quantify brain abnormalities relative to typical controls.

To generate normative EEG values, we perform meticulous EEG preprocessing on 24-h recordings—artifact removal (e.g., eye blinks, muscle noise), filtering, and referencing—to ensure clean data. Two manual expert reviews of the files to identify potential SEA in line with the previously published protocols (Kamondi et al., 2024; Port et al., 2019). To define normal limits, the density, amplitude, duration, and spatial distribution of these events can be quantified across all control recordings. Statistically, normal values are represented by upper tolerance limits (the 95th percentile) of spike density (events per hour) computed from the control group. Normative modeling is applied to further refine these thresholds by accounting for age, sex, sleep state, and recording montage, variables known to influence EEG background and transient patterns. Additionally, spectral power and band ratios (delta, theta, alpha, beta, gamma) are used to construct normative baselines, against which epileptiform spikes produce transient deviations.

#### 2.4.2 In-ward protocol

Participants will be admitted to a dedicated video-EEG monitoring unit for three consecutive days and nights.

- Continuous video-EEG: 34-channel scalp EEG (Micromed Morpheus, 10–20 system) will be recorded throughout admission, with peri-orbital leads for sleep staging.
- Memory paradigms: Three parallel versions of the RAVLT will be administered on consecutive evenings (7 pm).
  ○ Immediate recall (five learning trials) and delayed recall at 30 min.
  ○ Morning recall at ∼9 am to assess overnight retention.

- Sleep analysis:
  ○ Visual staging of 20-s epochs according to AASM criteria (Moser et al., 2009).
  ○ Artifact annotation in 4-s segments.
  ○ Automated slow and fast spindle detection with Individual Adjustment Method (Bódizs et al., 2009).
  ○ Quantification of spindle density, duration, amplitude, and frequency over frontal (slow spindles) and centro-parietal (fast spindles) leads, in N2 and N3, and combined N2+N3.
  ○ Power spectral analysis of N2 and N3 for slow-wave sleep characteristics.
- Serial blood sampling: Evening and morning samples across the 3-day stay to estimate circadian patterns of plasma tau and Aβ.

#### 2.4.3 Data extraction and mechanistic analyses

For each participant, we will derive:

- SEA density and temporal distribution across nights and days.
- Within-subject variability in RAVLT learning, 30-min retention, and overnight consolidation.
- Sleep architecture metrics and spindle parameters.
- Day–night and day-to-day changes in plasma tau/Aβ.

SEA density will be correlated with memory performance, spindle metrics, and biomarker levels, as assessed using mixed-effects models that account for repeated measures. Comparisons will be made between SEA-rich and SEA-poor epochs and between days with high vs low hyperexcitability burden. Group-specific patterns (e.g., MCI vs ASD vs ADHD) will be examined.

### 2.5 Data management and quality assurance

A detailed data management plan governs:

- Standardized naming conventions and metadata.
- Secure storage of pseudonymized data on institutional servers with routine backup.
- Separation of personal identifiers from research data; access limited to authorized staff.
- Harmonized SOPs for acquisition and preprocessing of EEG, MRI, and biomarker data.

Quality control steps include:

- Visual inspection of MRI segmentations and fMRI registrations.
- Inter-rater agreement for EEG interpretation (SEA detection) and sleep staging is formally evaluated using Cohen’s κ coefficient (with 95% confidence intervals) before consensus adjudication. Periodic calibration sessions are conducted throughout the study to maintain consistency in scoring across raters, and periodic auditing of neuropsychological scoring is conducted.
- Data-integrity milestones (EEG, neuroimaging) with open-source anonymized libraries planned for selected datasets.

Pseudo-anonymized datasets and analysis scripts will be made available to qualified investigators upon reasonable request, in line with ethical and legal frameworks, and will support future meta-analyses.

### 2.6 Statistical analysis

#### 2.6.1 Cross-sectional analyses

Data from all diagnostic groups will be pooled into a single multivariate dataset and analyzed using machine learning and dimensional reduction techniques to extract cross-diagnostic patterns of neural and behavioral variance. The model will test for latent factors reflecting shared E/I imbalance across disorders, while *post hoc* analyses identify disorder-specific deviations. Statistical adjustments will account for confounders such as age, medication, and cognitive status.

Primary outcome will be the (Torenvliet et al., 2023) incidence and distribution of SEA, and fMRI hyperactivity in each diagnostic group relative to controls. Secondary outcomes will include relationships between hyperexcitability and (a) structural atrophy and white matter integrity; (b) functional connectivity within and between default mode, salience, and attention networks; (c) CSF and plasma tau/Aβ levels; and (d) domain-specific cognitive performance.

- Incidence of hyperexcitability (primary outcome):
  ○ Logistic regression models comparing SEA and fMRI-positive status between groups (MCI, ASD, ADHD vs HC), adjusting for age, sex, education, and disease duration where applicable.
  ○ Results will be reported as odds ratios with 95% confidence intervals.

- Associations with cognition, structure, and connectivity (secondary outcomes):
  ○ Within each group, participants will be classified as hyperexcitability-positive vs negative (see section “2.4.1 Participant selection and extraction of normative values”).
  ○ For continuous outcomes (neuropsychological scores, cortical thickness/volume, DTI measures, functional connectivity indices, blood-based biomarker concentrations), group comparisons will use *t*-tests or Mann–Whitney U tests depending on normality (Shapiro-Wilk).
  ○ For categorical variables, chi-square tests will be used.
  ○ Multivariable linear models with covariates (age, sex, education, disease duration, mood/anxiety scores) will assess independent associations.
  Holm–Bonferroni corrections will be used to control for multiple comparisons.

- Cross-modal relationships:
  ○ Correlation and regression analyses will examine relationships between spike density and fMRI hyperactivity.
  ○ Multimodal latent variable models or clustering approaches will be applied exploratorily to define hyperexcitability phenotypes.

#### 2.6.2 Mechanistic analyses

- Linear mixed-effects models will relate within-subject variation in SEA density to RAVLT performance, sleep spindle parameters, and plasma tau/Aβ levels, with random intercepts for participants.
- Connectivity changes (morning vs evening, SEA-rich vs SEA-poor epochs) will be assessed with paired tests and network-based statistics.
- Mediation models will explore whether sleep spindle disruption mediates the effect of SEA on overnight memory consolidation.

Effect sizes, confidence intervals, and model diagnostics will be reported systematically.

**FIGURE 2:**
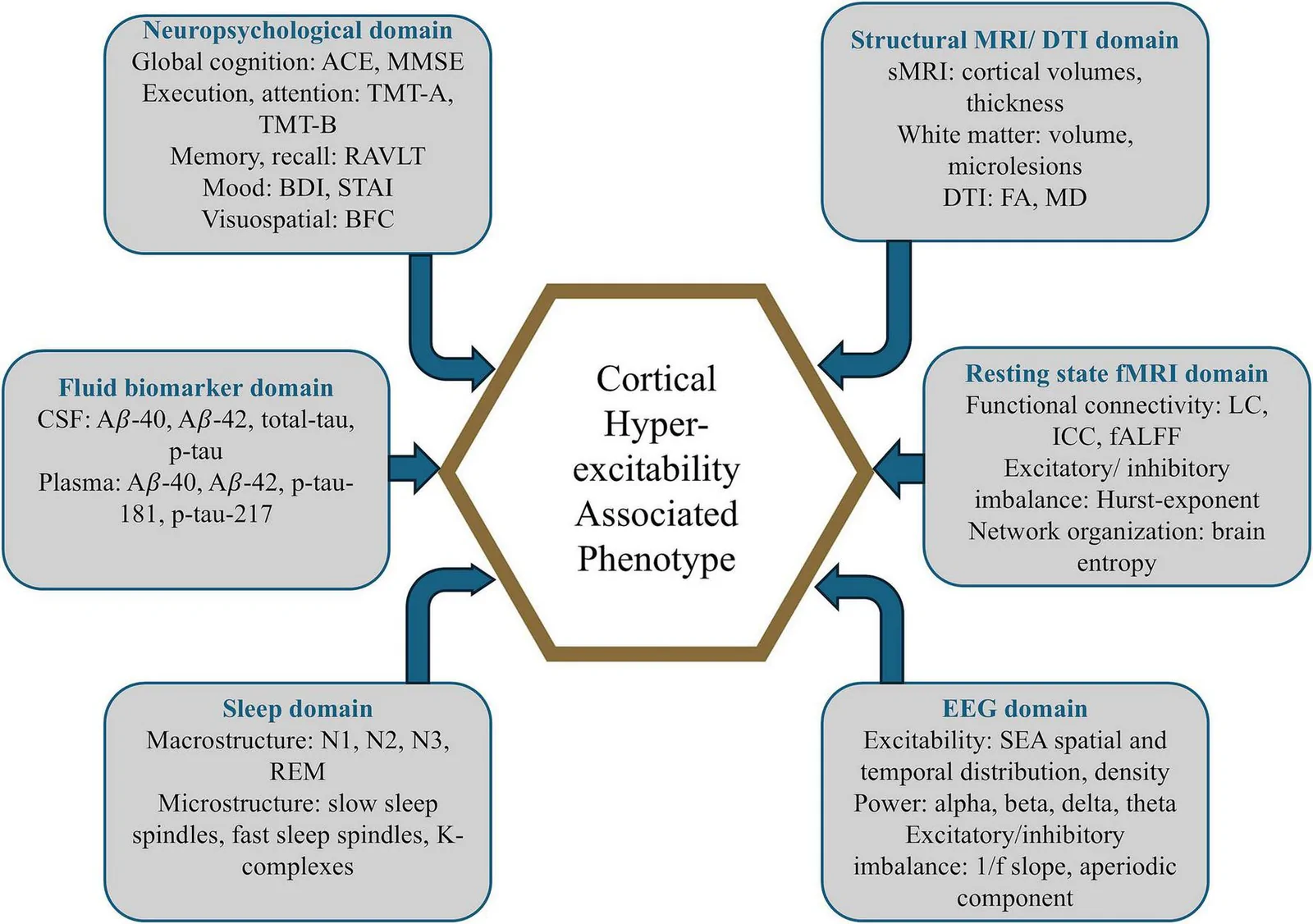
Multimodal biomarker domains integrated in the LENDÜLET protocol. ACE, Addenbrooke Cognitive Examination; MMSE, Mini-Mental State Examination; TMT, Trail-Making Test; RAVLT, Rey Auditory Verbal Learning; BDI, Beck Depression Inventory; STAI, Spielberger State and Trait Anxiety Questionnaire; BFC, Benson Figure Copy; Aβ, amyloid-beta; REM, rapid eye movement sleep; MRI, magnetic resonance imaging; sMRI, structural magnetic resonance imaging; fMRI, functional magnetic resonance imaging; DTI, diffusion tensor imaging, FA, fractional anisotrophy; MD, mean diffusivity; LC, local correlation; ICC, intrinsic connectivity; fALFF, fractional amplitude of low frequency fluctuations; SEA, subclinical epileptiform activity.

The entire analysis pipeline is highlighted in Figure 2.

## 3 Expected results and implications

This protocol outlines a comprehensive, multimodal approach to characterizing the association between cortical hyperexcitability and cognitive decline across neurocognitive disorders with high vulnerability to epileptic activity, namely MCI on the AD continuum, ASD, and ADHD. By integrating ambulatory and in-ward EEG, resting-state fMRI, structural MRI, detailed neuropsychology, sleep metrics, and tau/amyloid biomarkers, the LENDÜLET Neurocognitive Research Project seeks to move beyond descriptive associations and toward a mechanistic understanding of how SEA and network hyperactivity affect cognition and disease trajectories.

### 3.1 Positioning within existing literature

Previous work in AD and MCI has demonstrated that SEA is more prevalent than previously recognized and is associated with faster cognitive decline (Horvath et al., 2020; Kamondi et al., 2024; Vossel et al., 2026). Similarly, in ASD and ADHD, converging data from EEG, fMRI, and TMS studies point to altered excitation–inhibition balance, resting-state hyperconnectivity or hypoconnectivity in large-scale networks, and increased seizure risk (Beare et al., 2017; Hull et al., 2017; Konrad and Eickhoff, 2010; Liu et al., 2022; Salpekar, 2018; Vilela et al., 2022). However, existing studies have typically focused on single modalities, heterogeneous samples, or narrow diagnostic categories, and have rarely incorporated sleep-dependent memory consolidation or AD-related proteinopathies.

The present study will address these gaps in several ways:

- It applies a unified multimodal protocol across four groups (MCI, ASD, ADHD, HC), enabling direct comparison of hyperexcitability prevalence and signatures across disorders and relative to healthy aging.
- It combines EEG-defined SEA and fMRI-defined network hyperactivity, providing a cross-modal view of hyperexcitability at both fast electrophysiological and slower hemodynamic timescales.
- It embeds hyperexcitability within a broader framework of structural volumetry, white matter integrity, network connectivity, sleep architecture, sleep spindles, and tau/amyloid burden, thereby situating SEA and hyperactivity in the context of neurodegeneration and systems-level network reorganization.
- It includes an in-ward mechanistic substudy with repeated within-subject measures, enabling stronger inference about temporal relationships between hyperexcitability, sleep dynamics, and memory consolidation.

By integrating these elements, the LENDÜLET project will be well-positioned to test the overarching hypothesis that cortical hyperexcitability is not merely an epiphenomenon but a mechanistically relevant substrate linking disrupted network dynamics to cognitive decline.

### 3.2 Conceptual implications: hyperexcitability as a transdiagnostic mechanism

**FIGURE 3:**
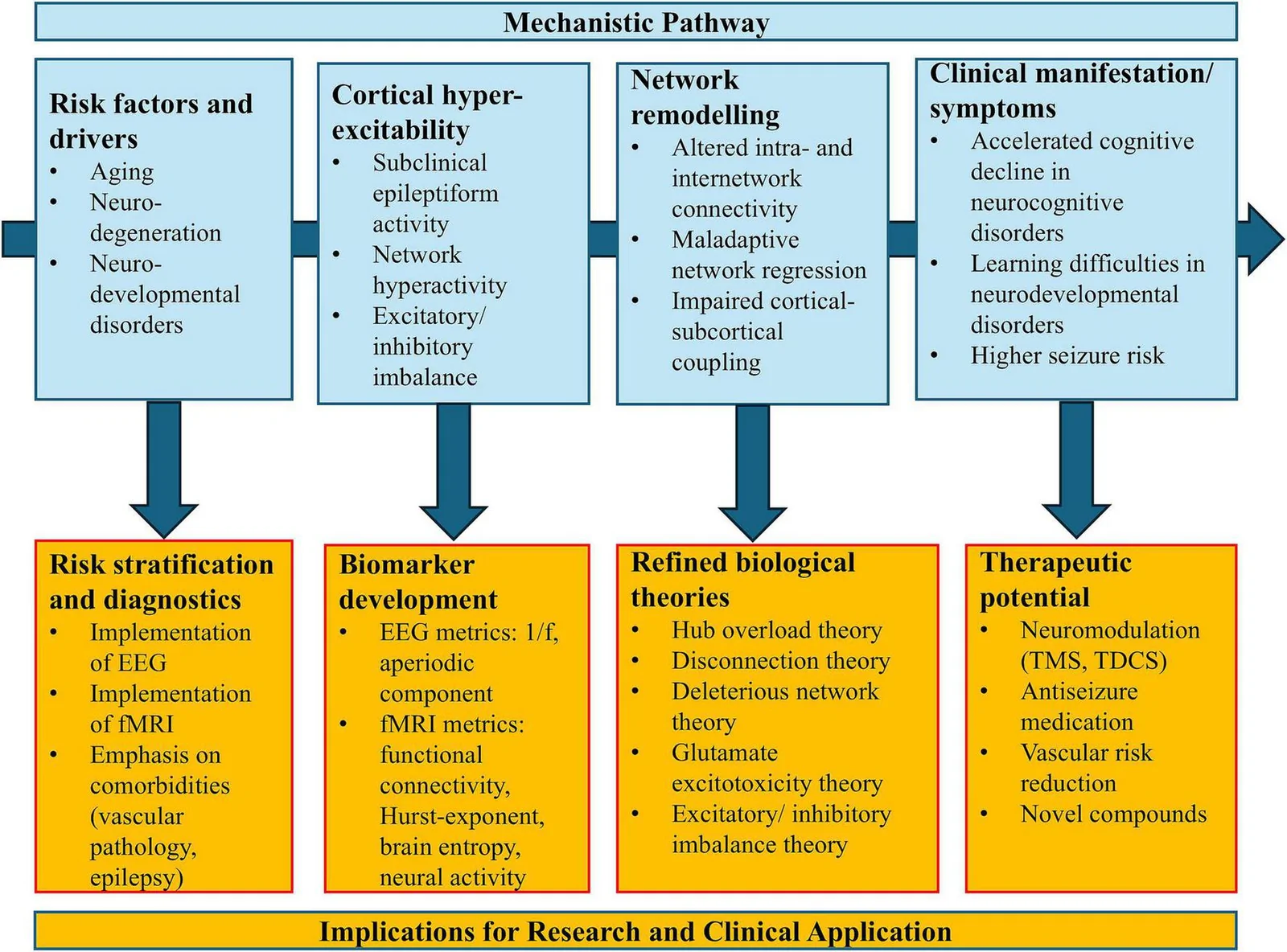
A comprehensive overview of the proposed mechanistic connection between neural changes related to hyperexcitability and their applicaability as implication for future biological, clinical and therapeutic experiments. EEG, electroencephalography; fMRI, functional magnetic resonance imaging; TMS, transcranial magnetic stimulation; TDCS, transcranial direct current stimulation.

If our hypotheses are confirmed, the findings will support the notion that cortical hyperexcitability is a transdiagnostic mechanism cutting across traditional categorical diagnoses. Several conceptual implications follow and are summarized in Figure 3:

- Shared hyperexcitability phenotype across neurocognitive disorders. Elevated SEA incidence and resting-state fMRI hyperactivity in MCI, ASD, and ADHD, relative to healthy controls, would support the presence of cortical hyperexcitability as a transdiagnostic neurophysiological phenotype. Importantly, such findings would not imply that identical pathophysiological mechanisms underlie hyperexcitability across these disorders. Rather, they would suggest that distinct genetic, developmental, and neurodegenerative processes may converge on a common manifestation of disrupted excitation–inhibition balance and network dysfunction, while differing in their underlying molecular drivers, spatial distribution, temporal evolution, and contribution to cognitive impairment (Sohal and Rubenstein, 2019). Consequently, any commonality identified by this study should be interpreted as convergence at the systems-neuroscience level rather than evidence of a uniform disease mechanism.
- Network-level vulnerability. Associations between hyperexcitability and altered connectivity within the DMN, SN, and AN may clarify why specific networks are repeatedly implicated in both seizure susceptibility and cognitive dysfunction. For example, hyperactivity in posterior DMN hubs (precuneus, posterior cingulate) might simultaneously promote epileptiform discharges and disrupt memory-related network communication (Stam, 2014).
- Link between hyperexcitability and neurodegeneration. Demonstrating that hyperexcitability co-localizes with specific patterns of cortical thinning and white matter damage, particularly in medial temporal and parietal regions, would strengthen the hypothesis that recurrent SEA and network overactivity signal structural injury as well. In the MCI/AD continuum, associations with unfavorable tau/Aβ profiles would further support a bidirectional relationship between excitability and proteinopathy (Tanaka et al., 2025; Targa Dias Anastacio et al., 2022).
- Role of sleep and memory consolidation. Findings that hyperexcitability is associated with disrupted sleep architecture, altered N2/N3 power spectra, and reduced or disorganized sleep spindles, together with impaired overnight verbal memory consolidation, would provide a mechanistic bridge between epileptiform activity and long-term cognitive decline. This would be consistent with the view that sleep-dependent memory consolidation is particularly vulnerable to abnormal neuronal synchrony and that nocturnal SEA may exert disproportionate cognitive impact (Li et al., 2022; Slutsky, 2024).
- Cross-modal concordance. Evidence of concordance between EEG-detected SEA and fMRI hyperactivity at the individual level would validate the use of complementary modalities for detecting hyperexcitability and might enable future stratification strategies that rely on whichever modality is more feasible or sensitive in a given setting (Von Wegner et al., 2018).

It should also be emphasized that the present study is not designed to establish that cortical hyperexcitability plays an identical mechanistic role across MCI, ASD, and ADHD. Instead, the multimodal framework is intended to identify both shared and disorder-specific characteristics of hyperexcitability. Subsequent analyses will therefore examine whether associations between SEA, functional network alterations, structural changes, sleep physiology, and cognitive performance differ between diagnostic groups. These comparisons will help determine whether cortical hyperexcitability represents a common downstream manifestation of distinct disease processes or reflects disorder-specific mechanisms with partially overlapping neurophysiological signatures.

### 3.3 Implications for future research

The project is primarily observational, but it is explicitly designed to generate hypotheses and tools for future interventional studies. Key research implications include:

#### 3.3.1 Biomarker development and validation

The multimodal dataset will allow derivation of a candidate biomarker panel of cortical hyperexcitability, combining:

- SEA incidence, density, and spatial distribution from 24-h and video-EEG recordings
- fMRI-based hyperactivity indices and aberrant connectivity patterns within DMN/SN/AN
- Structural MRI measures (regional cortical volumes/thickness, DTI metrics)
- Sleep spindle metrics and N2/N3 spectral characteristics
- Fluid biomarkers (CSF and plasma tau, Aβ40/42, Aβ42/Aβ40 ratio)

The cross-sectional arm will clarify which combinations of these features optimally discriminate hyperexcitability-positive from negative cases, and which best predict cognitive performance. The mechanistic arm will reveal which metrics track short-term fluctuations in excitability and cognition within individuals. Together, these findings can inform the design of composite scores or latent factors that may serve as more robust endpoints for future clinical trials (Toniolo et al., 2020).

#### 3.3.2 Target identification for interventional studies

By relating SEA density and network hyperactivity to specific cognitive domains, network nodes, and sleep features, the study may highlight concrete targets for interventional modulation, such as:

- Reducing nocturnal SEA in patients with MCI and early AD
- Normalizing DMN and salience network overactivity in ASD and ADHD
- Enhancing sleep spindle activity or stabilizing N2/N3 architecture in individuals with hyperexcitability-associated memory impairment

These targets can then be probed in controlled trials of anti-seizure medications (Vossel et al., 2021), neuromodulation approaches (e.g., TMS, tDCS) (Chai et al., 2019), or behavioral interventions that improve sleep quality and circadian regulation.

#### 3.3.3 Design of precision medicine trials

The stratified, mechanistic data from LENDÜLET will support the design of precision trials by:

- Identifying a subgroup of MCI patients with clear hyperexcitability-positive signatures and high risk of rapid progression, who might particularly benefit from early anti-hyperexcitability interventions
- Defining EEG/fMRI-based inclusion criteria and surrogate endpoints for proof-of-concept studies
- Providing quantitative benchmarks for expected changes in SEA density, network activity, and sleep measures that could be used to power future interventional trials

#### 3.3.4 Methodological contributions and data sharing

The project’s standardized acquisition and preprocessing pipelines for EEG, MRI, and sleep data, together with open-source anonymized libraries planned for selected datasets, will facilitate methodological advancements, including:

- Development and validation of automated SEA detection tools optimized for dementia and neurodevelopmental populations
- Testing of advanced connectivity and graph-theoretical metrics to characterize hyperexcitability-related network reconfiguration
- Exploration of multimodal clustering or latent variable models to delineate excitability-related endophenotypes that transcend traditional diagnostic boundaries

These resources may support future meta-analyses and collaborative efforts across centers, accelerating the translation of hyperexcitability research into clinical practice.

### 3.4 Clinical implications

Although the present study is not interventional, its results may have several near- and medium-term implications for clinical practice.

#### 3.4.1 Risk stratification and prognostication

In older adults with MCI or early AD, the systematic use of 24-h EEG and resting-state fMRI is not yet standard (Frisoni et al., 2024). If this project confirms that SEA and network hyperactivity robustly predict faster cognitive decline and more aggressive structural/biomarker profiles, this would support:

- Incorporating targeted EEG (particularly sleep EEG) into the diagnostic work-up for MCI patients with rapid decline, atypical features, or high clinical suspicion of unrecognized epileptiform activity (Horváth et al., 2018a)
- Considering EEG and fMRI markers when counseling patients and caregivers about prognosis, especially in those with intermediate or ambiguous biomarker profiles
- Using hyperexcitability status to guide the intensity and frequency of follow-up, including neuropsychological monitoring and imaging

#### 3.4.2 Refining the role of anti-seizure medications

Current practice regarding prophylactic anti-seizure treatment in AD and related dementias is highly variable, partly due to limited evidence and lack of reliable markers for treatment selection (Bosco et al., 2023; Williams et al., 2025). Should SEA and hyperactivity be linked to worse outcomes, future trials could evaluate:

- Whether treating SEA in the absence of clinical seizures improves cognition, slows decline, or normalizes sleep-related memory consolidation
- Whether specific anti-seizure drugs (e.g., those with favorable cognitive and sleep profiles) are better suited for hyperexcitability-positive MCI or AD patients
- Whether treatment response differs according to baseline network connectivity patterns, sleep spindle status, or tau/Aβ burden

Such evidence could eventually lead to more nuanced recommendations for anti-seizure therapy in older adults with cognitive impairment, moving beyond a purely seizure-centric paradigm (Talevi, 2022).

#### 3.4.3 Integration with sleep medicine and cognitive rehabilitation

The planned mechanistic analyses connecting SEA, sleep architecture, spindle dynamics, and memory consolidation highlight opportunities for interdisciplinary care:

- Sleep assessments (including polysomnography or extended EEG) could be more systematically integrated into the evaluation of patients with cognitive complaints, particularly those with suspected hyperexcitability
- Behavioral and pharmacological strategies that improve sleep continuity, increase slow-wave activity, or enhance spindle expression might be prioritized in hyperexcitability-positive patients, even in the absence of overt insomnia
- Cognitive rehabilitation programs may be adapted to leverage periods of lower excitability or better sleep quality (e.g., scheduling memory-intensive tasks when SEA burden is low, if such patterns are identifiable in individual patients)

#### 3.4.4 Relevance for ASD and ADHD across the lifespan

For adults with ASD and ADHD, the findings may help to:

- Identify individuals who, despite not meeting criteria for epilepsy, exhibit frequent SEA or network hyperactivity associated with attentional, learning, or behavioral deficits
- Inform decisions about when to pursue EEG or fMRI in routine practice, particularly in cases of unexplained cognitive deterioration or treatment-resistant symptoms
- Stimulate research into whether targeted modulation of hyperexcitability (pharmacological, neuromodulatory, or behavioral) can improve specific cognitive domains or reduce functional impairment in these populations

### 3.5 Strengths, limitations, and future directions

The strengths of the LENDÜLET project include its multimodal design, rigorous phenotyping, standardized protocols, and integration of both cross-sectional and mechanistic within-subject analyses. Nonetheless, several limitations should be acknowledged:

- As a single-center study, generalizability to other healthcare systems, ethnicities, and clinical settings may be limited; future multi-center replications will be important.
- The observational design precludes definitive causal inferences. Although the study is designed to identify transdiagnostic and disorder-specific patterns of cortical hyperexcitability, demonstrating similar electrophysiological or imaging signatures across diagnostic groups will not establish a common underlying pathophysiological mechanism. Differences in the biological origins, anatomical distribution, and functional consequences of hyperexcitability between MCI, ASD, and ADHD will require further investigation in longitudinal and interventional studies. The selection of participants for the in-ward substudy is enriched for hyperexcitability-positive individuals, which is appropriate for mechanistic analyses but may limit extrapolation to the full spectrum of each disorder.
- CSF sampling is restricted to MCI participants with clinical indication, potentially introducing selection bias in biomarker analyses; however, parallel plasma measures will partially mitigate this limitation.

Future work building on this protocol should include randomized controlled trials targeting hyperexcitability, studies extending the approach to other neurodegenerative and neuropsychiatric disorders, and longitudinal cohorts that track the evolution of excitability markers from preclinical to clinical stages. Incorporating genetic and molecular data (e.g., APOE genotype, polygenic risk scores, synaptic markers) could further refine mechanistic models and identify subgroups with particular vulnerability to excitability-related neurodegeneration.

## 4 Conclusion

The LENDÜLET Neurocognitive Research Project will provide the first systematic, multimodal characterization of cortical hyperexcitability across MCI, ASD, ADHD, and healthy aging within a unified protocol. By linking SEA and fMRI hyperactivity to cognitive performance, network connectivity, sleep-dependent memory consolidation, and tau/amyloid burden, the study aims to clarify whether hyperexcitability constitutes a central mechanistic pathway to cognitive decline. The resulting biomarker panel and mechanistic insights are expected to inform risk stratification, shape the design of future anti-hyperexcitability trials, and ultimately contribute to individualized prevention and treatment strategies for cognitive decline in older adults and across neurodevelopmental conditions.

## Footnotes

1
www.eufingers.com
